# Supplementary material for: Correction: Multimodal Holographic Microscopy: Distinction between Apoptosis and Oncosis
Source: PLoS One. 2015 May 1;10(5):e0127929. doi: 10.1371/journal.pone.0127929 (PMC4416793; doi:10.1371/journal.pone.0127929)
Supplement: S1 File — (PDF) [file pone.0127929.s001.pdf]

PLOS ONE

RESEARCH ARTICLE

Multimodal Holographic Microscopy: Distinction between Apoptosis and Oncosis

Jan Balvan[1,2], Aneta Krizova[2,3], Jaromir Gumulec[1,2], Martina Raudenska[1,2], Zbysek Sladek[4], Miroslava Sedlackova[5], Petr Babula[6], Marketa Sztalmachova[1,2], Rene Kizek[2,7], Radim Chmelik[2,8], Michal Masarik[1,2]*

1 Department of Pathological Physiology, Faculty of Medicine, Masaryk University, Brno, Czech Republic, **2** Central European Institute of Technology, Brno University of Technology, Brno, Czech Republic, **3** TESCAN Brno, s.r.o., Brno, Czech Republic, **4** Department of Morphology, Physiology, and Animal Genetics, Mendel University in Brno, Brno, Czech Republic, **5** Department of Histology and Embryology, Faculty of Medicine, Masaryk University, Brno, Czech Republic, **6** Department of Physiology, Faculty of Medicine, Masaryk University, Brno, Czech Republic, **7** Department of Chemistry and Biochemistry, Mendel University in Brno, Brno, Czech Republic, **8** Institute of Physical Engineering, Faculty of Mechanical Engineering, Brno University of Technology, Brno, Czech Republic

* masarik@med.muni.cz

CrossMark
click for updates

OPEN ACCESS

Citation: Balvan J, Krizova A, Gumulec J, Raudenska M, Sladek Z, Sedlackova M, et al. (2015) Multimodal Holographic Microscopy: Distinction between Apoptosis and Oncosis. PLoS ONE 10(3): e0121674. doi:10.1371/journal.pone.0121674

Academic Editor: Robert M. Hoffman, AntiCancer Inc., UNITED STATES

Received: October 3, 2014

Accepted: February 3, 2015

Published: March 24, 2015

Copyright: © 2015 Balvan et al. This is an open access article distributed under the terms of the Creative Commons Attribution License, which permits unrestricted use, distribution, and reproduction in any medium, provided the original author and source are credited.

Data Availability Statement: All relevant data are within the paper.

Funding: The financial support from CEITEC CZ.1.05/1.1.00/02.0068 and project for conceptual development of research organization (Faculty of Medicine, Masaryk university - ROZV/24/LF5/2014) is greatly acknowledged. Part of the work was carried out with the support of core facilities of CEITEC – Central European Institute of Technology under CEITEC-open access project, ID number LM2011020, funded by the Ministry of Education, Youth and Sports of the Czech Republic under the activity "Projects of major infrastructures for research,

Abstract

Identification of specific cell death is of a great value for many scientists. Predominant types of cell death can be detected by flow-cytometry (FCM). Nevertheless, the absence of cellular morphology analysis leads to the misclassification of cell death type due to underestimated oncosis. However, the definition of the oncosis is important because of its potential reversibility. Therefore, FCM analysis of cell death using annexin V/propidium iodide assay was compared with holographic microscopy coupled with fluorescence detection - "Multimodal holographic microscopy (MHM)". The aim was to highlight FCM limitations and to point out MHM advantages. It was shown that the annexin V+/PI– phenotype is not specific of early apoptotic cells, as previously believed, and that morphological criteria have to be necessarily combined with annexin V/PI for the cell death type to be ascertained precisely. MHM makes it possible to distinguish oncosis clearly from apoptosis and to stratify the progression of oncosis.

Introduction

Cell necrobiology is a rapidly developing field of cell biology that defines various modes of cell death pursuant to biochemical, morphological, and molecular changes accompanying distinct types of cell death including the tissue response [1].

Identification of the exact type of cell death following the cell injury is important for diagnostics, dose-response, and toxicological studies. It is extremely important to assess and interpret correctly the cellular response to severe injury including changes that occur before and after the cell death, because cell death changes could be the earliest signal of toxic reactions to a variety of drugs including the anticancer treatment. Cells can die through a number of different mechanisms *inter alia* by apoptosis, autophagy, necrosis, or oncosis. Nevertheless, two major

development and innovations". The funders had no role in study design, data collection and analysis, decision to publish, or preparation of the manuscript.

Competing Interests: The cooperation with commercial company (TESCAN) does not alter the authors' adherence to PLOS ONE policies on sharing data and materials.

types of cell death are accidental cell death and programmed cell death. When assessing the major effect of a particular therapeutic drug, it is essential to know which type of cell death is involved most in the drug response. If the main mechanism involved in the cell death is oncosis followed by necrosis, the cells lose membrane integrity and release their intracellular contents, which are often aggressive, proinflammatory, and cause damage to the surrounding tissue [2]. By contrast, apoptotic cells may not promote inflammation because they are usually ingested by phagocytes before releasing their intracellular contents [3]. An important biochemical event leading to oncosis /necrosis, as opposed to apoptosis, is a rapid decrease of intracellular ATP [4, 5]. The assessment of oncosis is frequently neglected, although it is an important pre-lethal phase that follows a serious cell injury and, unlike in necrosis, some mechanisms possibly exist for reversing the process [5].

Many changes typical for these two main types of cell death (accidental and programmed cell death) are detectable by flow-cytometry. Nevertheless, relying solely on the flow-cytometry could lead to the misclassification of the cell death type since—similarly as apoptotic cells—oncotic cells could exhibit external *phosphatidylserine* residues (PS) while maintaining membrane integrity. As a result, oncotic cells could display the annexin V+/PI− phenotype, formerly supposed to be specific of apoptotic cells [6, 7]. Similarly, the TUNEL assay is also known to be non-specific for apoptosis/oncosis differentiation [8–10]. Consequently, morphological criteria are considered the most reliable evidence of apoptosis [11, 12]. Characteristics of apoptosis, oncosis, and necrosis are summarized in Table 1.

In this paper, we present a methodology that can be used for the rapid assessment of cell viability and distinction of oncosis and apoptosis utilizing multimodal holographic microscope (MHM). MHM combines holographic microscopy with the well-known fluorescence microscopy. The employed holographic microscopy (HM) is based on an off-axis setup with an incoherent source. In contrast with the HM laser source, the incoherent HM source enables high-quality quantitative phase imaging free of speckles and parasitic interferences, comparable with the lateral resolution of conventional wide-field microscopes. Owing to the off-axis setup, only

Table 1. Characteristic features of apoptosis, oncosis, and necrosis.

Feature	Oncosis	Necrosis	Apoptosis
Cell size	increased (swelling)*	increased (swelling)*	reduced (shrinkage)*
Plasma membrane	intact* in the early phase; increased throughput depending on the phase of oncosis	disrupted*	intact*; altered orientation of lipids
Nucleus	Nucleus dilatation* and clumping of chromatine* reticular nucleolus	karyolysis* and caspase independent DNA fragmentation,lysis of nucleolus	nuclear chromatin condensation*; fragmentation of DNA into nucleosome size fragments, irregularity of nucleus*
Specific features	swelling of organelles; membrane blebs*	increasingly translucent cytoplasm*; swelling of ER and loss of ribosomes; swollen mitochondria with amorphous densities; lysosome rupture; plasma membrane rupture*; myelin figures	apoptotic bodies*; pseudopod retraction*; spherical shape of cells*
Energy balance	ATP depletion	ATP depletion	retained ATP production
PI/annexin V assay	annexin V+/ PI−*; annexin V+/PI+*	annexin V+/PI+*	annexin V+/ PI−*
Adjacent inflammation	frequent	frequent	rare

* indicates typical features of distinct cell death, easy observable by using MHM. ER—endoplasmatic reticulum; ATP—adenosine triphosphate; PI—propidium iodide. According to our results and [4, 5, 43].

doi:10.1371/journal.pone.0121674.t001

Table 2. Classification of holographic methods.

Method	Optical setup	Illumination source	Properties
Phase-shifting microscopes	In-line (zero angle between object and reference beam)	Low-coherence (halogen lamp, LED)	+ suppressed coherence noise
			+ coherence-gating effect
			+ lateral resolution of conventional microscopes
			– slow: 3 images for reconstruction
Digital holographic microscopes	off-axis (non-zero angle between object and reference beam)	High-coherence (laser)	– coherence noise
			– lateral resolution 2x worse than in conventional microscopes
			+ fast: 1 image/reconstruction
Coherence-controlled holographic microscopes	off-axis	low-coherence	+ suppressed coherence noise
			+ coherence-gating effect
			+ lateral resolution of conventional microscopes
			+ fast: 1image/reconstruction

doi:10.1371/journal.pone.0121674.t002

one hologram is needed for image reconstruction and very fast processes can be observed [13]. General characteristics of different holographic methods are summarized in Table 2. Holographic microscopy is a method of quantitative phase imaging. Providing intrinsic high contrast, phase images allow an easy segmentation of cells from the image background and monitor morphological and position changes over the time [14]. Fluorescence imaging is combined with holographic microscopy in a way that the focus plane in both methods is at the same position. This allows an easy transition between the two methods, imaging in the same conditions and nearly at the same time points. This unique combination enables a label-free observation of processes such as morphological and position changes preceding the cell death and a follow-up fluorescence verification of cell death types in one field of view using a single instrument.

In this study, PC-3 prostatic cell lines treated with plumbagin in concentrations exceeding IC50 were chosen as a model because our laboratory has a long-term experience in studying this ROS-generating agent and in characterizing this cell line [15–17]. In sum, the aim of this study was to highlight limitations of the flow-cytometry analysis of cell death and to point out advantages of MHM imaging. The hypothesis is that MHM is capable of differentiating between apoptosis and oncosis more accurately than flow-cytometry. Thus, we demonstrate a new possible application of this innovative microscopic technique.

Materials and Methods

Chemical and biochemical reagents

Ham's F12 medium, mycoplasma-free foetal bovine serum (FBS), penicillin/streptomycine and trypsine were purchased from PAA Laboratories GmbH (Pasching, Austria). Phosphate-buffered saline (PBS) was purchased from Invitrogen Corp. (Carlsbad, CA, USA). Ethylenediaminetetraacetic acid (EDTA), plumbagin and other chemicals of ACS purity were purchased from Sigma-Aldrich Co. (St. Louis, MO, USA), unless noted otherwise.

Cell cultures

Human PC-3 prostate cancer cells were used in this study. The PC-3 cell line was established from the prostatic adenocarcinoma (Grade 4) of a 62-year-old Caucasian male and derived

from a metastatic site in the bones. The PC-3 cell line was purchased from HPA Culture Collections (Salisbury, UK).

Cultured cell conditions

The PC-3 cells were cultured in Ham's F12 medium with 7% FBS. The medium was supplemented with penicillin (100 U/ml) and the cells were kept at 37°C in the humidified incubator with 5% CO2.

Plumbagin treatment

The stock solution of plumbagin was prepared in dimethylsulfoxide (DMSO) and diluted with the medium. Controls were added an equal volume of DMSO (final concentration $\leq$ 0.1%). The plumbagin treatment was initialized after the cells reached ~50% confluence. Previously, IC50 for plumbagin was determined as 1.50 μM using MTT. Thus, a treatment with 2 μM of plumbagin was used in this experiment to ensure the initiation of cell death.

Multimodal holographic microscopy

The design of the Multimodal Holographic Microscope (IPE BUT, TESCAN, Brno, Czech Republic) is based on the original concept of the Coherence-Controlled Holographic Microscope [18, 19].

Holographic microscopy was initiated after 2 h of the plumbagin treatment. Time-lapse monitoring was performed for 2 h (in total 4 h of plumbagin treatment) at a frame-rate of 1 frame/min. The cells were observed in flow chambers μ-Slide I Luer Family cat. Num. 80196 (Ibidi, Martinsried, Germany) in Ham's F12 medium. Nikon Plan 10×/0.3 and Nikon Plan Fluor 20×/0.5 objectives were used for both holographic and fluorescence observations. Interferograms for holography were taken using a CCD camera (XIMEA MR4021MC-VELETA). The fluorescence mode used a plasma light source (Sutter Instrument Lambda XL) and a CCD camera (XIMEA MR285MC-BH, 1392×1040px) was used to capture the images.

Holographic and fluorescence images were collected by custom software. In fluorescence image there is no need for other image processing; however, holographic raw data have to be numerically reconstructed. The numerical reconstruction is performed by the custom software where the established methods of the fast Fourier-transform [20] and phase unwrapping [21, 22] are implemented. The output from the software is an unwrapped phase image. This image has intrinsic high contrast and can be processed by an available image processing software.

We used particularly the ImageJ functions of *Thresholding* and *Analysed Particles*. At chosen time points of the time-lapse observation, the cells were segmented from the background, a threshold value for the segmentation being 0.21 rad (0.05 pg/pixel). Each cell was controlled visually, and cells in contact were separated manually. Measurements of cell surface, cell dry mass and mean cell dry mass followed.

TEM visualization of ultrastructure. PC-3 cells were gently harvested by repetitive pipetting and spun down (2000 rpm, 5 min.). Briefly, the cells were fixed with 3% glutaraldehyde in a cacodylate buffer for 2 hours and washed three times for 30 minutes in 0.1 M cacodylate buffer. Then they were fixed with 0.02 M OsO4 dissolved in 0.1 M cacodylate buffer, dehydrated in alcohol, and infiltrated with acetone and No. 1 Durcuptan mixture overnight. On the following day, the cells were infiltrated with No. 2 Durcuptan mixture, embedded and polymerized. Ultrathin sections (90 nm, Ultramicrotome LKB, Bromma, Stockholm, Sweden) were transferred onto grids covered with a Formvar membrane (Marivac Ltd., Halifax, Canada). 2% uranyl acetate and Reynolds solution were used for contrast staining. The sections were viewed in the transmission electron microscope (Morgagni 268, FEI Europe B.V., Eindhoven

Netherlands). Software AnalySIS (Soft Imaging System, GmbH, Münster, Germany) was used for a picture analysis of the cell ultrastructure.

Annexin V/propidium iodide flow-cytometry

Double-staining with fluorescein isothiocyanate (FITC)/propidium iodide (PI) was performed using the annexin V-FLUOS-staining kit (Roche Applied Science) according to the manufacturer's protocol in order to determine percentages of viable, apoptotic, and necrotic cells following the exposure to plumbagin (2 uM). Briefly, the cells were harvested by repetitive pipetting and washed two times with PBS (centrifuged at 2000 rpm for 5 min). Then they were re-suspended in 100 μl of the annexin V-FLUOS labelling solution and incubated for 15 min. in the dark at 15–25°C. Annexin V-FITC binding was detected by flow cytometry (Partec GmbH, Münster, Germany) (Ex = 488 nm, Em = 533 nm, FL1 filter for annexin V-FLUOS and FL3 filter for PI). The data were analyzed using the FloMax software version 2.5 (Partec GmbH, Münster, Germany).

Results

Development of the multimodal holographic microscope

The employed multimodal holographic microscope was developed in cooperation between researchers from the Brno University of Technology and the TESCAN Brno Company. The MHM design (IPE BUT, TESCAN, Brno, Czech Republic) is based on the concept of the Coherence-Controlled Holographic Microscope (CCHM) described in [19]. This novel optical setup of CCHM overcomes drawbacks of the previous concept [18], while preserving all benefits and enabling multimodal imaging.

The holographic mode setup is shown in Fig. 1. It is based on the Mach-Zehnder-type interferometer. The light from the source (S) passes through the collector lens (CL) and is divided by the beam splitter (BS) into two separated optical paths—object and interferometer reference arm. The beams are directed by mirrors (M). Both arms consist of condenser (C), objective (O) and tube lens (TL). In the reference arm, a diffraction grating (DG) is placed. The output lenses (OL) focus the beams onto the output plane. There the object beam and the reference beam recombine and create an interference fringes pattern (hologram), which is captured by the camera (D).

The amplitude and the phase image are reconstructed numerically from the hologram. The process of the numerical reconstruction is based on the fast Fourier transform methods [20]. For time-lapse sequences, the image processing discussed in [23] is applied. The entire image reconstruction and image processing are performed by our own software. The resulting phase image can be used for classic image processing and analysis, e.g. for segmentation that defines what is the background and what is the cell in the image. From the phase image, various other visualization modalities can be easily obtained by simple numerical calculations [24]. Here we used a simulated differential interference contrast (simulated DIC) that was calculated as a 1D gradient of the quantitative phase image.

Flow-cytometry analysis of cell death

First, non-stained cells (control) were analyzed using flow-cytometry to set the annexin V /PI gating regions (Fig. 2A). Consequently, non-treated cells and cells treated with plumbagin for 3 h were analyzed (Fig. 3B and C, resp.). Four different phenotypes were distinguished: (a) annexin V−/PI− (lower left quadrant, Q3); estimated as viable cells; (b) annexin V+/PI− (lower right quadrant, Q4); usually estimated as apoptotic cells, but probably could contain larger

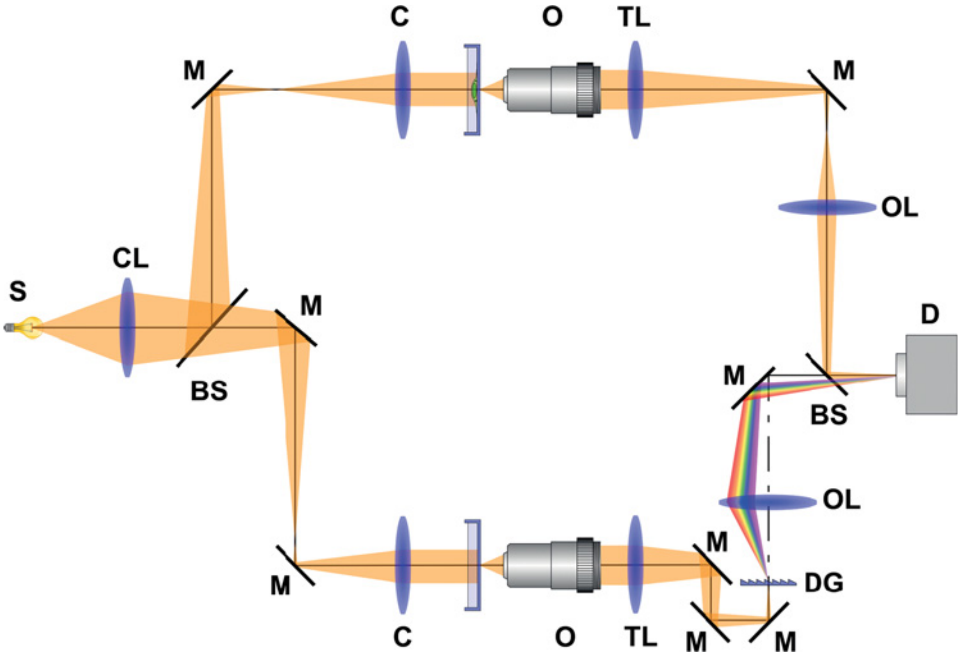

Fig 1. Holographic mode setup in Multimodal holographic microscope is based on the Mach-Zehnder-type interferometer. The light is divided into two separate optical paths—object arm and interferometer reference arm. Both arms consist of condenser (C), objective (O) and tube lens (TL). In the reference arm, a diffraction grating (DG) is placed. The object beam and the reference beam recombine in the output plane and create interference fringes pattern, which is captured by the camera (D). S—source; CL—collector lens; BS—beam splitter; M—mirror; C—condenser; O-objective; TL—tube lens; DG—diffraction grating; OL—output lens; D—detector.

doi:10.1371/journal.pone.0121674.g001

quantities of oncotic cells; (c) annexin V−/PI+ (upper left quadrant, Q1); fragments of damaged cells; (d) annexin V+/ PI+ (upper right quadrant, Q2); late apoptotic and necrotic cells.

Compared to the non-treated cells, the fraction of annexin V+/PI− cells increased distinctly to 30.55% (compared to 4.50% in the non-treated sample), and the fraction of annexin V+/PI + increased up to 7.75% (compared to 1.70% in the non-treated sample). Furthermore, the studied cells were back-gated in the dot plot with the forward scatter/side scatter parameters of these treated cells. As a result of the back-gating, two different annexin V+/PI− populations can be seen (gated in green colour) (Fig. 2C, lower dot plot, arrows R1 and R2). Therefore, additional re-gating was performed. As a result, annexin V+/ PI− and lower FSC (region R1, supposed apoptosis) formed 9.51% of cells, and annexin V+/ PI− and higher FSC (region R2, supposed oncosis) formed 13.22% of cells (gating process not shown).

Time-lapse holographic microscopy

Subsequently, the same plumbagin-treated PC-3 cell line was observed using MHM. Attention was focused on changes in the cellular morphological features including cell shape, cell mass, cell spreading area, and typical structures, which could be seen in the injured cells. Using the time-lapse analysis, we identified 32% of cells which increased their volume and showed increasing blebs in the plasma membrane during the time-lapse analysis (designated as "increase size" in Fig. 2D), 24% of cells, which decreased their volume and showed apoptotic bodies (designated as "decrease size"), and 44% of cells with the unchanged volume during the 2 h of monitoring. From the phase images, we also measured cell surface mass over the time-lapse

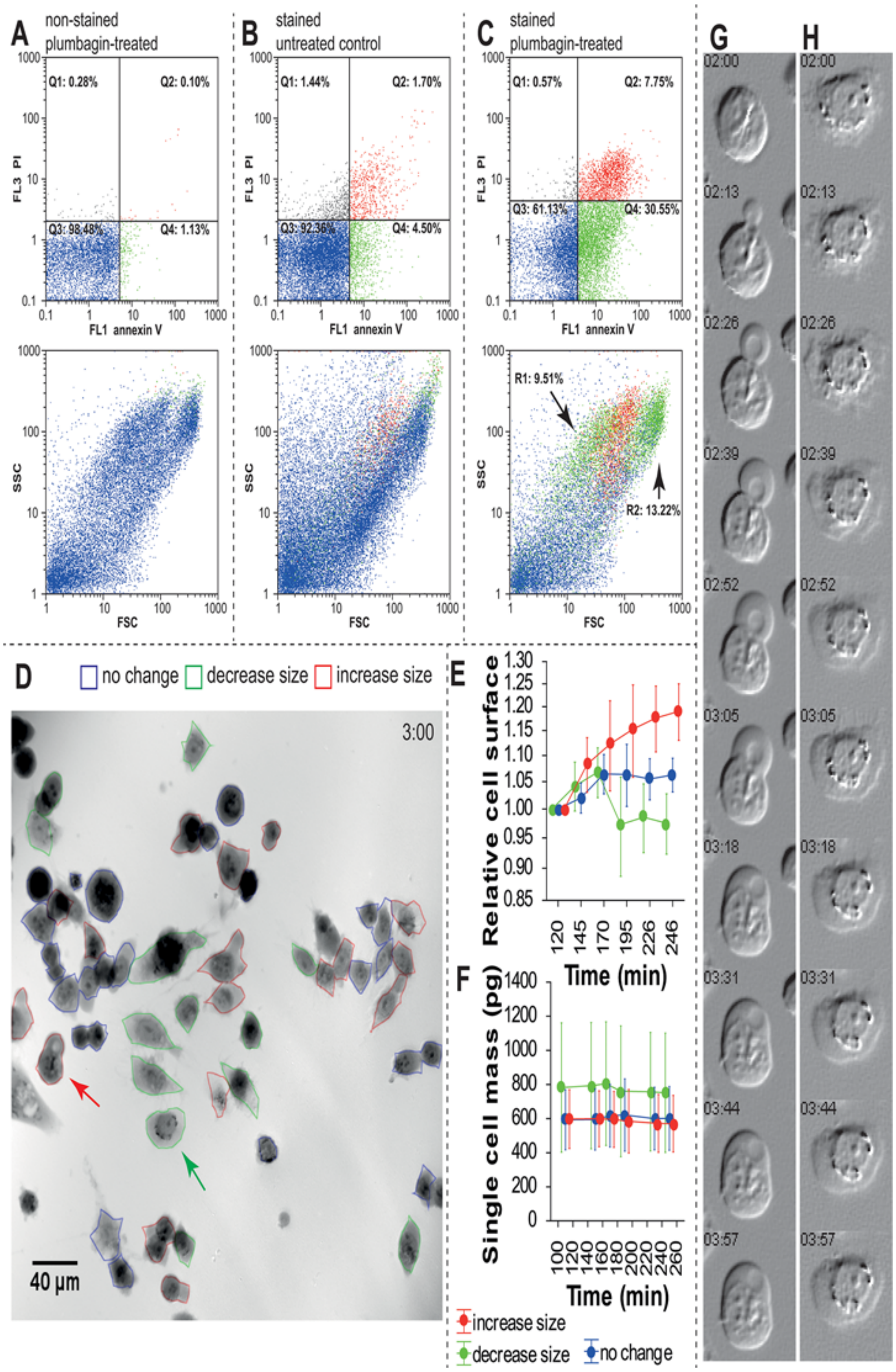

Fig 2. Differences in cell death estimation between flow cytometry and holographic microscopy. A. Plumbagin treatment, no annexin V/PI staining, used for gating set-up. Upper dot plot indicates annexin V/PI fluorescence, lower dot plot indicates FSC/SSC of these cells colour-coded according to gating regions. **B.** Annexin V/PI staining, untreated cells. 92% are double negative for staining. **C.** Annexin V/PI staining after 3 h of the experiment. See increased populations of annexin V-positive (green gating) and double positive (red staining). In FSC/SSC scatter plot, arrows indicate two populations (gating regions) of annexin V+/PI– cells: (R1) smaller cells (lower FSC) and (R2) larger cells (higher FSC). See the Results section for details. **D.** Multimodal holographic microscope, phase image. 10 × magnification 3 h after 2 μM plumbagin treatment. Red-outlined cells show size increase and oncotic phenotype, green-outlined cells show surface area decrease and apoptotic phenotype, blue-outlined cells show no changes during 2 h monitoring. For typical

morphological criteria of oncotic/apoptic cells see (Table 1) **E**. Relative change of cell surface in individual cells (relative to initial time point). Colour coding of individual cells is based on (D). **F**. Mass of individual cells in pg. Note a significantly higher mass of the "decrease-size" cell population. **G**. Time-lapse of typical oncotic "increase size" cell indicated by red arrow in (D), simulated digital interference microscopy **H.** Time-lapse of typical "decrease size" apoptotic cell indicated by green arrow in (D). Simulated digital interference microscopy. FSC—forward scatter, SSC—side scatter, PI—propidium iodide.

doi:10.1371/journal.pone.0121674.g002

observation (Fig. 2E-F). Accordingly, the surface area of the cells differed significantly between these three groups of cells, $F(2, 244) = 31.05$, $p < 0.001$, namely at later time points. With regard to cell mass, the results are in accordance with the assumption that this parameter remains nearly unchanged over this short time in the cell cycle. 90% of the observed cells changed their mass by less than 7%, $F(5, 244) = 0.03$, $p = 0.99$. In contrast, the mass of cells was significantly higher in the "decrease size" group of cells as compared to the "no change" and "increase size" groups, $F(5, 244) = 4.68$, $p = 0.01$; the mass of the "decrease size" cells was on average 1.3-fold higher (Fig. 2F).

By contrast to oncosis or necrosis, which are associated with the cell swelling and increased cell volume, apoptosis is connected with the loss of cell volume during the cell shrinkage (for detailed characterization of morphological changes of each cell death type see the below section of this text). Regarding the fact, 2×3 two way contingency tables were created to compare HM with the flow-cytometry results (no size change in HM vs. double-negative cells in FCM; "increase size" vs. annexin V+/PI+ population, and "decrease size" vs. annexin V+/PI− population). There was a significant difference between the assays; the proportion of the "increase size" cells was significantly higher when determined by holographic microscopy, $\chi^2 = 18.043$, $p = 0.0001$ (Table 3). Because two annexin V+/PI− populations (green) were found on the FSC/SSC dot-plot by back gating, another chi-squared test was performed. Unlike in the previous testing, the "increase size" was compared with the annexin V+/PI+ population (Q2) plus R2 population (see above for details) and the "decrease size" included only the R1 gating region. Although there was still a significant difference, the proportional difference between the assays was not that profound and hence the p-level was higher (Table 3).

Assessment of apoptotic, oncotic, and necrotic cells morphology

Consequently, morphology of the cells after the plumbagin treatment was described using MHM, light microscopy, and verified at ultrastructural level using transmission electron microscopy (TEM). In agreement with the previous chapter, three populations of cells were observed according to size changes. Apart from cellular shrinking or swelling, other characteristics typical of distinct cell deaths were observed in MHM (see Table 1). In the oncotic cells, an intact plasma membrane with cytoplasmic blebs, nuclear chromatin clumping, and nucleus dilatation were detected. The formation of cytoplasmic blebs is well apparent in the time lapse (Fig. 2G).

Some major morphological features connected with necrosis were observed too, including multiple and large cytoplasmic blebs, translucent cytoplasm, cell swelling ended by cell membrane disruption and nucleus dilatation (Fig. 3). In contrast, the shrinking group of cells exhibited characteristics typical of apoptosis: spherical shape of the cells, chromatin condensation, nuclear membrane irregularity and indeed the formation of multiple apoptotic bodies considered as an advanced stage of apoptosis (Fig. 3A, second column). Based on these characteristics, typical morphological criteria visible in MHM are illustrated in scheme (Fig. 3B). By using the fluorescence mode of MHM, we detected annexin V staining in all observed types of cell deaths

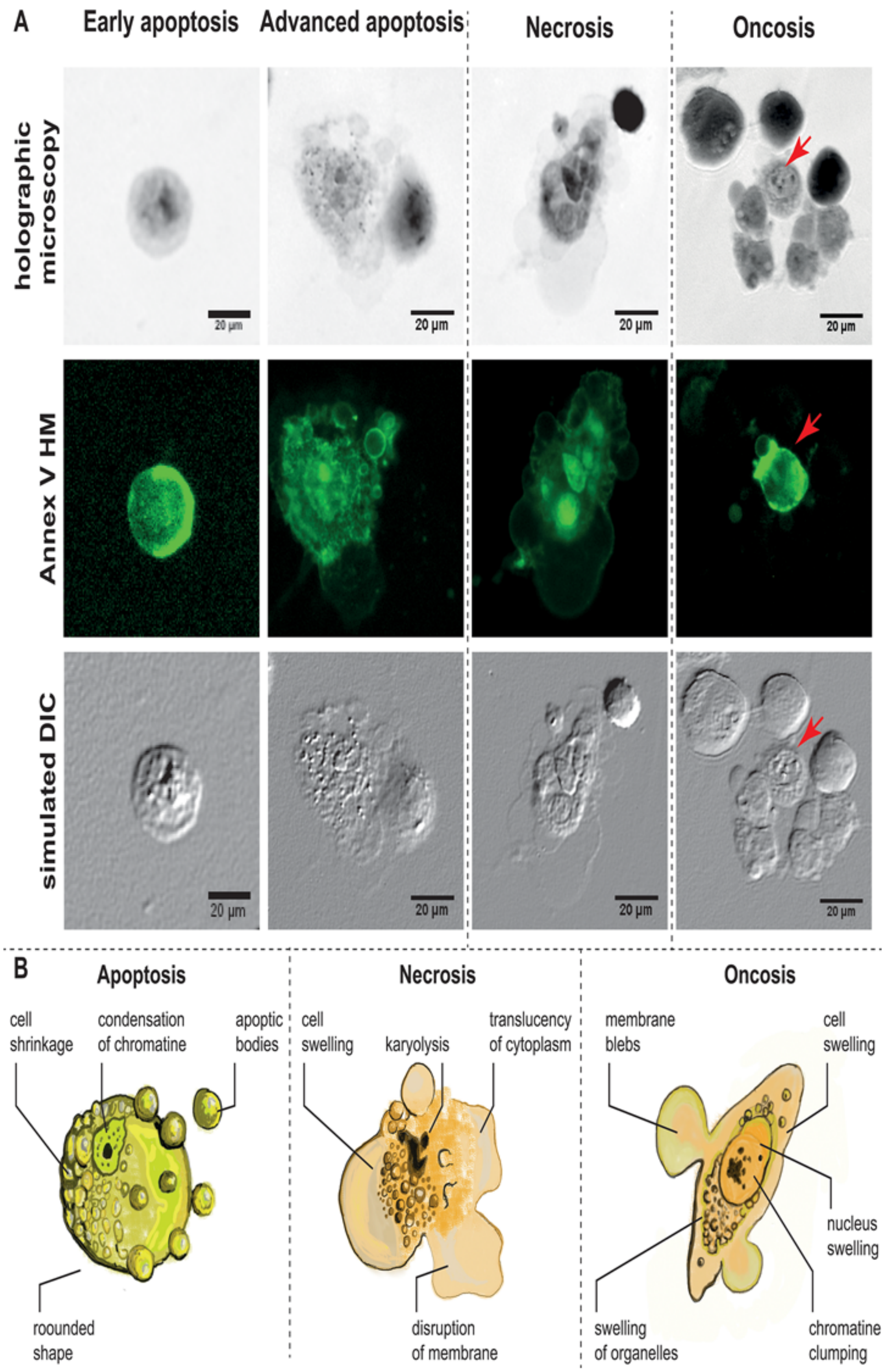

Fig 3. Morphology of Apoptotic, necrotic and oncotic cells. A. Characteristic apoptotic, necrotic and oncotic cells in multimodal holographic microscope, simulated DIC (differential interference contrast). 20 × magnification was used in MHM. Annexin V staining for the verification of cell membrane alteration. Red arrow indicates annexin V-positive "advanced" oncotic cell. Apoptotic cells displayed in initial step (left) with the typically round-shaped cells and in advanced step with the formation of apoptotic bodies. **B.** Scheme of typical apoptotic, necrotic and oncotic cells. Typical characteristics visible by MHM phase image. For a detailed description of the characteristic features of apoptotic, necrotic, and oncotic cells, see Table 1.

doi:10.1371/journal.pone.0121674.g003

Table 3. Two-way contingency table of the observed cell death frequencies based on morphological criteria and flow-cytometry analysis.

Technique	Cell classification			χ2	p level
	normal	increase size	decrease size		
phase images from MHM	44	32	24	-	-
Flow-cytometry†	61	8	31	18.04	0.0001
Flow-cytometry, adjusted for oncosis‡	61	13	21	10.85	0.0044

† For "flow cytometry", annexin V+/ PI+ cells are designated as "increase size", and annexin V+/PI– cells are designated as "decrease size". Compared to phase images from MHM.

‡ for „flow cytometry, adjusted for oncosis", annexin V+/ PI– cells gated by lower FSC (i.e. smaller cells, arrow R1 in Fig. 2C) are designated as "decrease size" (reflect "true apoptosis"), and summed annexin V+/PI+ and annexin V+/PI– cells gated by higher FSC (i.e. larger cells, arrow R2 in Fig. 2C) are designated as "increase size" (reflect necrosis and oncosis, resp.). Compared to phase images from MHM. PI—propidium iodide, FSC—forward scatter.

doi:10.1371/journal.pone.0121674.t003

(oncosis, necrosis, and apoptosis as well; Fig. 3A). In early apoptotic cells, annexin V positivity was detected, namely nearby the cell membrane (Fig. 3A, first column).

All mentioned morphological characteristics were corroborated by light microscopy (data not shown) and at the ultrastructural level by using TEM. Further cell death features observable only with TEM were found. *Inter alia*, dilatation of endoplasmic reticulum (ER) and Golgi apparatus, condensation of mitochondria followed by their swelling and rupture, formation of cytoplasmic vacuoles, swollen and ruptured lysosomes, lysis of nucleolus and karyolysis in oncotic/necrotic cells were apparent (Fig. 4 and unpublished data). In apoptotic cells, significant nuclear fragmentation was observed (Fig. 4).

According to our results, MHM is not able to recognize ultrastructural details of cells (mitochondria, ER, etc.), but is more suitable for detecting membrane blebs and apoptotic bodies. The recognition of membrane blebs and apoptotic bodies in MHM was even better than with TEM.

Assessment of oncosis progression using MHM

Multimodal holographic microscope combines holographic imaging with fluorescent imaging. The unique device makes it possible to combine the assessment of main morphologic features with annexin V/PI staining using a single instrument in one field of view. Thus, this technique was employed for an in-depth analysis of oncotic cells.

Cells with the characteristic morphological criteria of oncosis exhibit various conditions of the PI/annexin V staining. We identified the following variants of oncotic cells: (1) small membrane blebs, higher mass of cells and annexin V−/PI− staining; (2) more frequent/larger plasmatic blebs, gradual loss of spindle cell shape, clumping of chromatin, annexin V+/ PI − staining; (3) more frequent/larger plasmatic blebs, lower mass of cells, significant swelling of nucleus with a still distinguishable nuclear membrane and chromatin condensation, annexin V +/PI+; (4) annexin V+/PI+ combined with the homogenization of nuclear structure, cell membrane disruption with a partial extrusion of cell contents, and other morphological criteria of necrosis (Fig. 5). Based on these apparently gradual morphological and staining characteristics, a staging of oncosis was suggested as follows: normal cells/oncosis transition, early oncosis, late oncosis, oncosis/necrosis transition.

Discussion

Methods of quantitative phase imaging are rapidly developing at present. Although their principles had been known for many years, the methods were not widely used until computer technology recorded a sufficient progress.

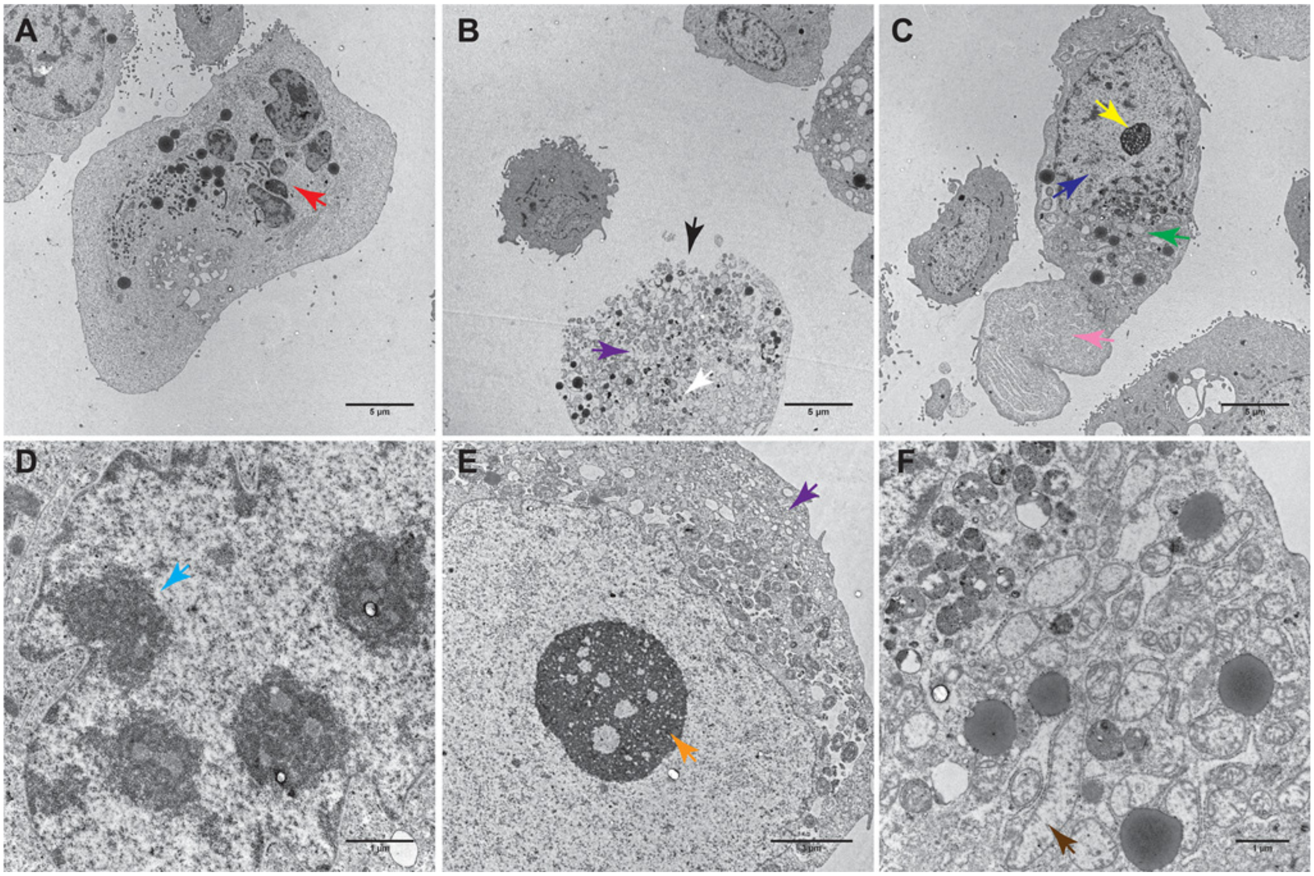

Fig 4. Characteristic apoptotic, necrotic and oncotic cells in transmission electron microscope (TEM). A. Apoptotic cell, overall view, 2800 ×. **B**. necrotic cell, 2800 ×. **C**. oncotic cell, 2800 ×, **D**. Detail of apoptotic cell nucleus, 5600 ×. **E**. Detail of necrotic cell, 14000 ×. **F**. Detail of oncotic cell cytoplasm, 11000 × magnification. Red arrow—nuclear fragmentation. Black arrow—rupture of plasmatic membrane. White arrow—karyolysis. Yellow arrow—reticular nucleolus. Dark blue arrow—dilatation of nucleus. Green arrow—dilatation of ER and Golgi. Pink arrow—cytoplasmic bleb. Light blue arrow—chromatin condensation. Violet arrow—formation of cytoplasmic vacuoles. Orange arrow—initial lysis of nucleolus. Brown arrow—mitochondrial swelling.

doi:10.1371/journal.pone.0121674.g004

Phase-shift ing microscopes [25–27] provide images with the suppressed coherence noise and lateral resolution comparable to that of conventional microscopes. They use low-coherence sources and owing to that, the coherence gating effect can be exploited. Disadvantage is the fact that three images, each with a different induced phase shift, have to be captured for one numerical reconstruction. Therefore, mechanical displacements have to be used and the acquisition rate is limited.

On the other hand, digital holographic microscopes with the off-axis setup and laser sources [28–30] allow a fast acquisition rate because only a single image is needed for the reconstruction. However, coherence noise is higher, lateral resolution is lower, and the coherence gating effect cannot be used.

The employed MHM is based on the Coherence-Controlled Holographic Microscope (CCHM) [18] and links advantages of the both previously developed methods of holographic microscopy. Moreover, MHM enables to combine holographic microscopy with other techniques [19].

MHM allows quantitative phase contrast imaging combined with other proven methods such as fluorescence microscopy. The method constitutes an overlap of traditional microscopy

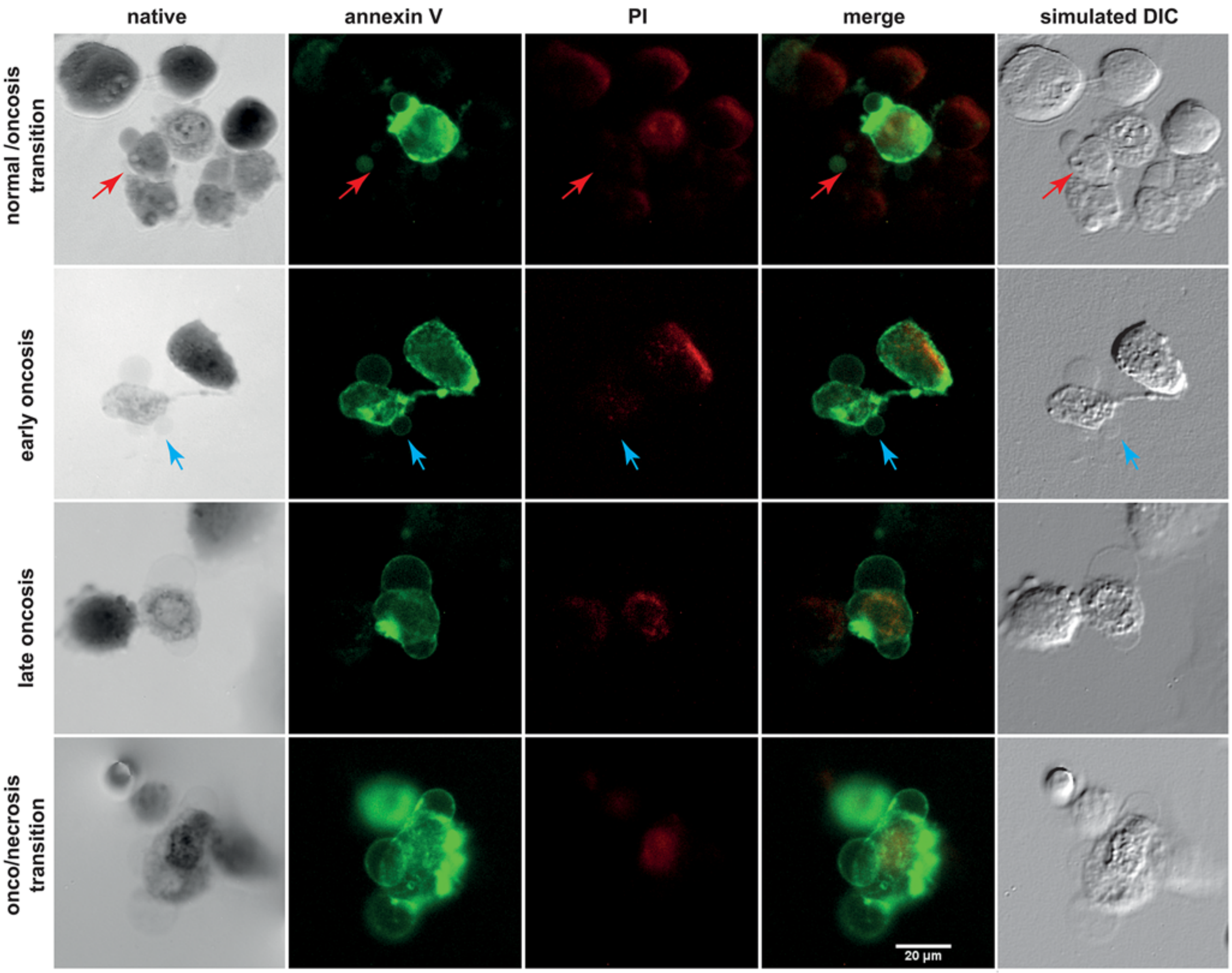

Fig 5. Estimation of oncosis progression by multimodal holographic microscopy. Annexin V (green) and propidium iodide (PI, red) staining. Initial step of oncosis (first row, red arrow) is annexin V–/PI– and thus distinguishable only by native morphology, see typical cytoplasmic bleb in the phase image. This causes false-negativity by flow-cytometry. Second, early oncotic cells feature larger blebs and are annexin V+/PI–. Late oncosis is double positive for staining, with no apparent karyolysis. Advanced oncosis/necrosis transition is typical by double-positive staining and karyolysis.

doi:10.1371/journal.pone.0121674.g005

techniques. Information in the phase image provides values of optical path delay caused by the observed cell. These values are directly proportional to the cell dry mass, originally published in [31, 32], and therefore, an evaluation of cell mass changes is possible [33–36]. Owing to this, holographic microscopy and similar techniques of quantitative phase imaging are useful tools for research in cell biology, such as cell dynamics studies, cancer research [37], intracellular mass transport [38], membrane fluctuations [39], cell cycle [33], cell growth [34] and others [40]. Digital holographic microscopy is highly suitable for studying dynamic processes, which are important for the analysis of living cells. Its potential for utilization in basic and applied research is promising. Accordingly, the monitoring of green- or red- fluorescent protein-expressing cells appears to be very promising using this instrument. Such a technique allows the visualization of cellular processes such as cell cycle, cell death, nuclear cytoplasma dynamics of viable cells in real time, which is expedient as compared with the use of artificial fluorescent staining techniques [41, 42].

In this study, we focused on distinguishing apoptosis from oncosis as a relevant example of the possible MHM use. Many authors use the annexin V/PI assay for the detection and quantification of apoptosis but often do not validate the type of cell death by assessing morphological features. In healthy cells, phosphatidylserine residues (PS) occur on the inner side of the cytoplasmic membrane. PS is externalized during apoptosis or, as our results showed, also in early oncosis by flipping from the inner to the outer layer of plasma membrane to enable the binding of annexin V. Propidium iodide (PI) detects cells with a disrupted plasma membrane by nuclear DNA staining. Unfortunately, using the conventional annexin V/PI assay in flow-cytometry, it is not always simple to differentiate apoptosis from oncosis [6], because, as our results showed, oncosis and apoptosis can occur simultaneously in the quadrant Q4 (annexin V+/PI −) of the dot plot. Thus, significant difference between the estimation of cell death using FCM and morphological criteria was observed. Therefore, it was not surprising that the estimation of cell processes using FCM and MHM differed significantly and led to the overestimation of the apoptotic population using FCM.

Interestingly, the back gating of flow-cytometry fluorescence data gave us unique results. Back gating of the annexin V+/PI− population to the forward scatter/side scatter dot plot visualized two sharply demarcated different populations: (1) a population of smaller annexin V + cells (lower FSC parameter, marked as R1 in Fig. 2), and (2) a population of larger annexin V + cells. In addition, back gating of the annexin V+/PI+ population to FSC/SSC dot plot brought us an additional information regarding the cell size: these double-positive cells ranged in size between the two annexin V+ populations. In accordance with the knowledge of the size of apoptotic, necrotic, and oncotic cells, this back gating allowed us to distinguish the populations of small apoptotic cells from the large oncotic cells. This back gating also confirmed that (double-positive) necrotic cells are larger than apoptotic cells and demonstrated that the necrotic cell population is smaller than that of the oncotic cells. These findings were taken into account for a further comparison of FCM and MHM. This time, however, the population of oncotic cells was added to necrotic cells and not to apoptotic cells. This "FCM adjustment for oncosis" resulted in markedly reduced differences between FCM and MHM; the difference was still significant though. Consequently, morphological criteria were shown to be the most reliable evidence of apoptosis. Apart from the cellular shrinking, other characteristics typical of apoptotic cells were apparent in MHM, e.g. nuclear irregularity, spherical cell shape, chromatin condensation, formation of multiple apoptotic bodies, and annexin V positivity, particularly near the cell membrane. Predominant characteristics typical of oncotic cells observable in MHM were cellular swelling, nuclear chromatin clumping, and formation of organelle-free blebs in the cytoplasmic membrane. These typical morphological criteria of distinct cell deaths were in compliance with results obtained from the transmission electron microscope (TEM) and also with the previous studies [4, 5, 43]. Thus, MHM can be considered a sufficient tool to estimate the cell fate.

Because oncosis and apoptosis can occur in response to the same drug, it is very important to distinguish carefully between these different kinds of cell death when testing new therapeutic drugs [44, 45]. Great emphasis is put also on the dynamic assessment of the prevalent effect of a particular therapeutic drug at different concentrations [46], because many dose–response studies have shown that cells exposed to lower concentrations of drugs such as cisplatin, etoposide, arsenic trioxide or doxorubicin, mostly exhibit the morphological characteristic of apoptosis but at higher doses, their morphology is typical for oncosis [4, 44, 47]. Dynamic (real-time) monitoring of treated cells is another important MHM application. The real-time monitoring makes it possible to observe particular cell death phases including the final fate of cells after the treatment, which is a significant advantage as compared with the common light microscopy providing only a kind of the end-point analysis. It was demonstrated that even if the

cells are driven to oncosis, there could be some mechanisms to reverse the process [4, 5], which can be easily captured by real-time MHM monitoring. Thus, MHM might provide a new valuable insight into cell biology.

Author Contributions

Conceived and designed the experiments: JB MR JG MM. Performed the experiments: JB AK M. Sedlackova M. Sztalmachova ZS PB. Analyzed the data: JG RK MR MM PB. Contributed reagents/materials/analysis tools: MM RK RC. Wrote the paper: JB AK JG MR ZS MM.

References

1. Wlodkowic D, Skommer J, Darzynkiewicz Z. Cytometry in Cell Necrobiology Revisited. Recent Advances and New Vistas. Cytometry A. 2010; 77A: 591–606.
2. Haanen C, Vermes I. Apoptosis and inflammation. Mediators Inflamm. 1995; 4: 5–15. PMID: 18475609
3. Rock KL, Kono H. The inflammatory response to cell death. Annual Review of Pathology: Mechanisms of Disease. Palo Alto: Annual Reviews; 2008. p. 99–126.
4. Weerasinghe P, Buja LM. Oncosis: An important non-apoptotic mode of cell death. Experimental and Molecular Pathology. 2012; 93: 302–308. doi: 10.1016/j.yexmp.2012.09.018 PMID: 23036471
5. Trump BF, Berezesky IK, Chang SH, Phelps PC. The pathways of cell death: Oncosis, apoptosis, and necrosis. Toxicologic Pathology. 1997; 25: 82–88. PMID: 9061857
6. Lecoeur H, Prevost MC, Gougeon ML. Oncosis is associated with exposure of phosphatidylserine residues on the outside layer of the plasma membrane: A reconsideration of the specificity of the annexin V/Propidium iodide assay. Cytometry. 2001; 44: 65–72. PMID: 11309810
7. Krysko O, de Ridder L, Cornelissen M. Phosphatidylserine exposure during early primary necrosis (oncosis) in JB6 cells as evidenced by immunogold labeling technique. Apoptosis. 2004; 9: 495–500. PMID: 15192332
8. Graslkraupp B, Ruttkaynedecky B, Koudelka H, Bukowska K, Bursch W, Schultehermann R. In-situ detection of fragmented DNA (tunel assay) fails to discriminate among apoptosis, necrosis, and autolytic cell-death—A cautionary note. Hepatology. 1995; 21: 1465–1468. PMID: 7737654
9. Buja LM, Entman ML. Modes of myocardial cell injury and cell death in ischemic heart disease. Circulation. 1998; 98: 1355–1357. PMID: 9760287
10. Freude B, Masters TN, Kostin S, Robicsek F, Schaper J. Cardiomyocyte apoptosis in acute and chronic conditions. Basic Res Cardiol. 1998; 93: 85–89. PMID: 9833169
11. Mahdi EJ, Alshahrani AM, Abdulsatar AA, Mahdi JG. Morphological evaluation of apoptosis induced by salicylates in HT-1080 human fibrosarcoma cells. Journal of Microscopy and Ultrastructure. 2014; 2: 20–27.
12. Jolly PD, Smith PR, Heath DA, Hudson NL, Lun S, Still LA, et al. Morphological evidence of apoptosis and the prevalence of apoptotic versus mitotic cells in the membrana granulosa of ovarian follicles during spontaneous and induced atresia in ewes. Biology of Reproduction. 1997; 56: 837–846. PMID: 9096863
13. Chmelik R, Slaba M, Kollarova V, Slaby T, Lostak M, Collakova J, et al. Chapter 5—The Role of Coherence in Image Formation in Holographic Microscopy. In: Wolf E, editor. Progress in Optics. Amsterdam: Elsevier; 2014. p. 267–335.
14. Dunn GA, Zicha D. Phase-shifting interference microscopy applied to the analysis of cell behavior. Symposia of the Society for Experimental Biology. 1993; 47: 91–106. PMID: 8165581
15. Gumulec J, Balvan J, Sztalmachova M, Raudenska M, Dvorakova V, Knopfova L, et al. Cisplatin-resistant prostate cancer model: Differences in antioxidant system, apoptosis and cell cycle. International Journal of Oncology. 2014; 44: 923–933. doi: 10.3892/ijo.2013.2223 PMID: 24366574
16. Holubova M, Axmanova M, Gumulec J, Raudenska M, Sztalmachova M, Babula P, et al. KRAS NF-kappaB is involved in the development of zinc resistance and reduced curability in prostate cancer. Metallomics. 2014; 6: 1240–53. doi: 10.1039/c4mt00065j PMID: 24927480
17. Masarik M, Gumulec J, Hlavna M, Sztalmachova M, Babula P, Raudenska M, et al. Monitoring of the prostate tumour cells redox state and real-time proliferation by novel biophysical techniques and fluorescent staining. Integrative Biology. 2012; 4: 672–684. doi: 10.1039/c2ib00157h PMID: 22592803
18. Kolman P, Chmelik R. Coherence-controlled holographic microscope. Optics Express. 2010; 18: 21990–22003. doi: 10.1364/OE.18.021990 PMID: 20941100

19. Slaby T, Kolman P, Dostal Z, Antos M, Lost'ak M, Chmelik R. Off-axis setup taking full advantage of incoherent illumination in coherence-controlled holographic microscope. Optics Express. 2013; 21: 14747–14762. doi: 10.1364/OE.21.014747 PMID: 23787662
20. Kreis T. Digital holographic interference-phase measurement using the fourier-transform method. J Opt Soc Am A Opt Image Sci Vis. 1986; 3: 847–855.
21. Goldstein RM, Zebker HA, Werner CL. Satellite radar interferometry—two-dimensional phase unwrapping. Radio Sci. 1988; 23: 713–720.
22. Ghiglia DC, Pritt MD. Two-dimensional phase unwrapping: theory, algorithms, and software: Wiley; 1998.
23. Zikmund T, Kvasnica L, Tyc M, Krizova A, Collakova J, Chmelik R. Sequential processing of quantitative phase images for the study of cell behaviour in real-time digital holographic microscopy. J Microsc. 2014: 117–125. doi: 10.1111/jmi.12190 PMID: 25354652
24. Wang Z, Millet L, Chan V, Ding H, Gillette MU, Bashir R, et al. Label-free intracellular transport measured by spatial light interference microscopy. J Biomed Opt. 2011; 16.
25. Dubois F, Joannes L, Legros JC. Improved three-dimensional imaging with a digital holography microscope with a source of partial spatial coherence. Applied Optics. 1999; 38: 7085–7094. PMID: 18324255
26. Zhang T, Yamaguchi I. Three-dimensional microscopy with phase-shifting digital holography. Optics Letters. 1998; 23: 1221–1223. PMID: 18087480
27. Xu L, Miao JM, Asundi A. Properties of digital holography based on in-line configuration. Opt Eng. 2000; 39: 3214–3219.
28. Cuche E, Bevilacqua F, Depeursinge C. Digital holography for quantitative phase-contrast imaging. Optics Letters. 1999; 24: 291–293. PMID: 18071483
29. Carl D, Kemper B, Wernicke G, von Bally G. Parameter-optimized digital holographic microscope for high-resolution living-cell analysis. Applied Optics. 2004; 43: 6536–6544. PMID: 15646774
30. Shin D, Daneshpanah M, Anand A, Javidi B. Optofluidic system for three-dimensional sensing and identification of micro-organisms with digital holographic microscopy. Optics Letters. 2010; 35: 4066–4068. doi: 10.1364/OL.35.004066 PMID: 21124614
31. Davies HG, Wilkins MHF. Interference microscopy and mass determination. Nature. 1952; 169: 541–541. PMID: 14929230
32. Barer R. Refractometry and interferometry of living cells. J Opt Soc Am. 1957; 47: 545–556. PMID: 13429433
33. Girshovitz P, Shaked NT. Generalized cell morphological parameters based on interferometric phase microscopy and their application to cell life cycle characterization. Biomed Opt Express. 2012; 3: 1757–1773. doi: 10.1364/BOE.3.001757 PMID: 22876342
34. Popescu G, Park Y, Lue N, Best-Popescu C, Deflores L, Dasari RR, et al. Optical imaging of cell mass and growth dynamics. Am J Physiol Cell Physiol. 2008; 295: C538–C544. doi: 10.1152/ajpcell.00121.2008 PMID: 18562484
35. Rappaz B, Cano E, Colomb T, Kuehn J, Depeursinge C, Simanis V, et al. Noninvasive characterization of the fission yeast cell cycle by monitoring dry mass with digital holographic microscopy. J Biomed Opt. 2009; 14.
36. Shaked NT, Satterwhite LL, Rinehart MT, Wax A. Quantitative Analysis of Biological Cells Using Digital Holographic Microscopy, Holography, Research and Technologies. Rosen J, editor. Rijeka: InTech; 2011.
37. Janeckova H, Vesely P, Chmelik R. Proving Tumour Cells by Acute Nutritional/Energy Deprivation as a Survival Threat: A Task for Microscopy. Anticancer Research. 2009; 29: 2339–2345. PMID: 19528500
38. Boss D, Kuehn J, Jourdain P, Depeursinge C, Magistretti PJ, Marquet P. Measurement of absolute cell volume, osmotic membrane water permeability, and refractive index of transmembrane water and solute flux by digital holographic microscopy. J Biomed Opt. 2013; 18.
39. Rappaz B, Barbul A, Hoffmann A, Boss D, Korenstein R, Depeursinge C, et al. Spatial analysis of erythrocyte membrane fluctuations by digital holographic microscopy. Blood Cells Mol Dis. 2009; 42: 228–232. doi: 10.1016/j.bcmd.2009.01.018 PMID: 19324576
40. Popescu G. Quantitative phase imaging of cells and tissues. 1st ed. New York: McGraw-Hill Professional; 2011.
41. Hoffman RM, Yang M. Subcellular imaging in the live mouse. Nat Protoc. 2006; 1: 775–782. PMID: 17406307

42. Yamamoto N, Jiang P, Yang M, Xu MX, Yamauchi K, Tsuchiya H, et al. Cellular dynamics visualized in live cells in vitro and in vivo by differential dual-color nuclear-cytoplasmic fluorescent-protein expression. Cancer Res. 2004; 64: 4251–4256. PMID: 15205338

43. Edinger AL, Thompson CB. Death by design: apoptosis, necrosis and autophagy. Current Opinion in Cell Biology. 2004; 16: 663–669. PMID: 15530778

44. Gonzalez VM, Fuertes MA, Alonso C, Perez JM. Is cisplatin-induced cell death always produced by apoptosis? Mol Pharmacol. 2001; 59: 657–63. PMID: 11259608

45. Hinson JA, Roberts DW, James LP. Mechanisms of acetaminophen-induced liver necrosis. Handb Exp Pharmacol. 2010: 369–405. doi: 10.1007/978-3-540-79088-4_17 PMID: 20020374

46. Chanan-Khan A, Srinivasan S, Czuczman MS. Prevention and management of cardiotoxicity from antineoplastic therapy. J Support Oncol. 2004; 2: 251–266. PMID: 15328825

47. Zhu JB, Okumura H, Ohtake S, Nakamura S, Nakao S. The molecular mechanism of arsenic trioxide-induced apoptosis and oncosis in leukemia/lymphoma cell lines. Acta Haematol. 2003; 110: 1–10. PMID: 12975549
